# The multi-faceted nature of age-associated osteoporosis

**DOI:** 10.1016/j.bonr.2024.101750

**Published:** 2024-03-05

**Authors:** A.E. Smit, O.C. Meijer, E.M. Winter

**Affiliations:** a Department of Medicine, Division of Endocrinology, Leiden University Medical Center, Leiden, the Netherlands; b Einthoven Laboratory for Experimental Vascular Medicine, Leiden, the Netherlands; c Department of Medicine, Center for Bone Quality, Leiden University Medical Center, Leiden, the Netherlands

**Keywords:** Age-associated osteoporosis, Fracture risk, Osteoporosis etiology, Prevention strategies, Personalized care

## Abstract

Age-associated osteoporosis (AAOP) poses a significant health burden, characterized by increased fracture risk due to declining bone mass and strength. Effective prevention and early treatment strategies are crucial to mitigate the disease burden and the associated healthcare costs. Current therapeutic approaches effectively target the individual contributing factors to AAOP. Nonetheless, the management of AAOP is complicated by the multitude of variables that affect its development. Main intrinsic and extrinsic factors contributing to AAOP risk are reviewed here, including mechanical unloading, nutrient deficiency, hormonal disbalance, disrupted metabolism, cognitive decline, inflammation and circadian disruption. Furthermore, it is discussed how these can be targeted for prevention and treatment. Although valuable as individual targets for intervention, the interconnectedness of these risk factors result in a unique etiology for every patient. Acknowledgement of the multifaceted nature of AAOP will enable the development of more effective and sustainable management strategies, based on a holistic, patient-centered approach.

## Highlights

• Age-associated osteoporosis (AAOP) has a complex, multifactorial etiology.
• AAOP risk is modifiable through lifestyle and medication, even in the oldest old.
• An integrated AAOP disease model will enable personalized intervention strategies.

## Graphical abstract

**Figure:**
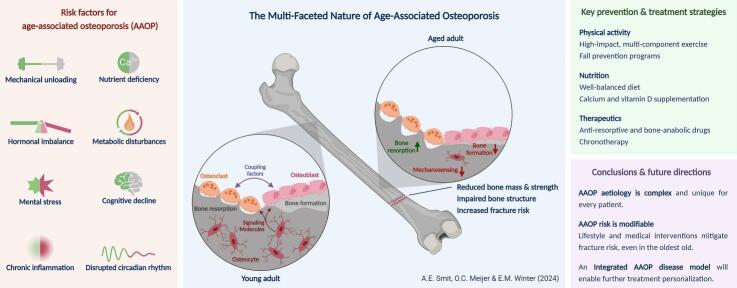

## 1 Introduction

**Fig. 1:**
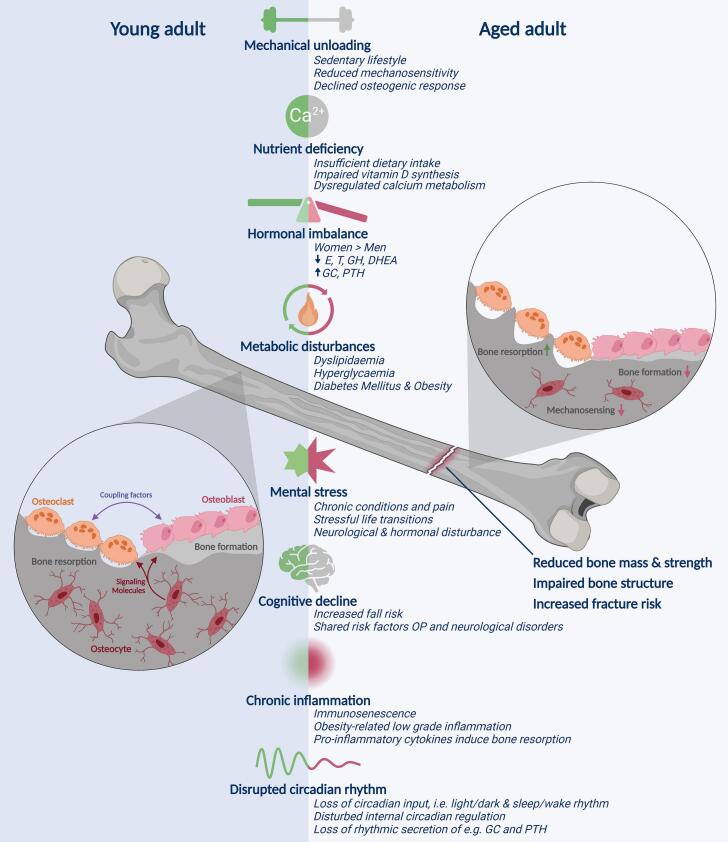
Age-related changes in intrinsic and extrinsic factors impact bone health, contributing to osteoporosis. These factors, whether independently or in combination, disrupt bone remodeling, resulting in diminished mass, strength, and an increased risk of fractures. E = estrogen, T = testosterone, GH = growth hormone, DHEA = Dehydroepiandrosterone, GC = glucocorticoid, PTH = parathyroid hormone, OP = osteoporosis.

Within our aging society, it is increasingly important to effectively prevent and treat age-related diseases. Osteoporosis (OP) is of particular concern. It is characterized by an increased fracture risk due to a decline in bone mass and/or quality, and affects over 35 % of elderly women and 12 % of elderly men worldwide (Salari et al., 2021). While aging is not a uniform process, the term elderly typically refers to individuals above the age of 65 (WHO, 2018). Osteoporotic fractures induce a high disease burden and their treatment drives health care costs. Therefore, early detection and efficient prevention strategies are a priority. A multitude of intrinsic and extrinsic risk factors for age-associated osteoporosis (AAOP) have been identified throughout the years (Fig. 1). While these have added value as individual risk factors, they are inevitably intertwined. This is demonstrated within two patient scenarios, revealing a unique AAOP etiology for each patient.

Scenario 1 **Preserving bone health during anti-inflammatory therapy**

The interplay of risk factors is illustrated by the following scenario of Mrs. Veenstra, a 69-year-old post-menopausal woman who is dependent on glucocorticoid (GC) medication for her polymyalgia rheumatica (PMR).

She presented with pain and stiffness in her neck, shoulders and pelvic girdle, particularly in the morning (Michet and Matteson, 2008). This pain had been present for over a year. Upon diagnosis, she exhibited elevated inflammatory markers, which, considering her symptom history, were likely already present for an extended duration. Although GC medications rapidly mitigated her symptoms, prolonged GC use likely accelerated the natural decline in bone mass she experienced since menopause.

Mrs. Veenstra is at high risk for osteoporotic fractures, due to her postmenopausal state and prolonged GC use. Furthermore, a period of increased inflammatory state may also have contributed negatively to her bone health.

The intervention plan should be aimed at preventing fractures while also managing PMR symptoms. Optimized intake of calcium and vitamin D and adequate physical exercise provide a low-cost and effective intervention strategy to improve bone mass in elderly. Notably, beneficial effects of regular exercise extend beyond bone health, potentially ameliorating PMR by improving muscle strength and curbing inflammation. In addition to lifestyle modifications, bone-protecting pharmacological agents can further improve bone health without exacerbating PMR symptoms. This medication should be advised depending on her fracture risk, taking GC dosage and her bone mineral density into account.

Alt-text: Scenario 1

Figure was created with BioRender.com.

## 2 Pathophysiology of osteoporosis

Healthy bone tissue is continually being remodelled. Old or damaged bone tissue is resorbed by osteoclasts, while osteoblasts deposit new organic bone matrix, consisting of collagen fibres, glycoproteins and proteoglycans (Hadjidakis and Androulakis, 2006; Sims and Martin, 2014). Finally, bone gains strength and stiffness through mineralization, which entails the deposition of calcium and phosphate ions as hydroxyapatite crystals (Murshed, 2018). Osteocytes, terminally differentiated osteoblasts that account for 90–95 % of all bone cells, coordinate the process (BONEWALD, 2007). In OP, bone resorption outpaces formation, resulting in a net loss of bone mass. In addition, changes in bone microarchitecture disturb the structural integrity, rendering bone brittle and fragile. The resulting weakened bone structure becomes prone to fractures, even from low-impact mechanical strain. This combination of a low bone mineral density (BMD) and an increased fracture risk entails the clinical definition of OP (Kanis, 1994). While most fractures occur after falling, the spine is particularly vulnerable to spontaneous osteoporotic fractures. This susceptibility arises from the high percentage of trabecular bone and the constant strain imposed by the body's weight on the spine (Chan et al., 2016).

Gender influences bone modeling and remodeling throughout life. During childhood, bone diameter expands through bone formation on the outer periosteal surface and resorption on the inner endosteal surface. During puberty, boys experience further expansion in bone diameter through continued periosteal growth, while girls predominantly gain bone mass trough endosteal growth (Lauretani et al., 2008). When bone mass reaches a plateau around the age of 25–30 years, men generally have larger bones with a wider diameter that can withstand more strain. Periosteal growth continues throughout life in both men and women. However, it is eventually outpaced by endosteal resorption, leading to a net loss in bone mass. Women experience a more pronounced loss, especially during the first postmenopausal years due to estrogen deficiency (Demontiero et al., 2012; Szulc et al., 2006). Men exhibit a slower, gradual decline, partly due to a higher rate of periosteal apposition as compared to women (Duan et al., 2001; Seeman, 2001). Bone loss persists well into old age for both genders. Data from the Framingham Osteoporosis study among individuals aged 67–90 revealed that over a 4-year period, women experienced BMD losses ranging from 3.4 % to 4.8 %, while men lost between 0.2 % and 3.6 %. As women typically undergo menopause at the age of 49–52 years, women in this study were presumably beyond their initial postmenopausal years (Morabia and Costanza, 1998). BMD loss was skeletal site dependent, with the radial shaft showing the highest rate of decline in both men and women (Hannan et al., 2000). Gender differences in bone size, diameter and BMD lead to varying fracture risks, with women over the age of 60 having an estimated lifetime risk of 44 %, compared to 25 % for men of the same age (Nguyen et al., 2007).

Beyond gender differences, twin and family studies indicate that hereditary factors determine the vast majority of variation in BMD (Brown et al., 2004; Hofer and Sliwinski, 2001; Mitchell et al., 2003; Videman et al., 2007). Genome-wide association studies (GWAS) have identified dozens of gene variants that influence BMD variation, and several of these are also associated with fracture risk (Estrada et al., 2012; Morris et al., 2019). For instance, variants of the gene for receptor activator of nuclear factor-kappa B ligand (RANKL) have been associated with have been associated with BMD in men (Hsu et al., 2006). Mutations in a single gene with a crucial role in bone remodeling can also induce skeletal fragility, often surpassing the impact of common variants identified through GWAS (Mäkitie and Zillikens, 2022; Robinson and Rauch, 2019). For instance, osteogenesis imperfecta patients suffer from low BMD and high susceptibility to fractures, in most cases caused by mutations in genes encoding for type I collagen*, COL1A1 and COL1A2* (Morello, 2018). Furthermore, genetic defects in Wnt/β-catenin signaling, i.e. loss-of-function mutations in *LRP5* and *WNT1*, lead to extremely low BMD, skeletal deformity and early onset osteoporosis (Mäkitie and Zillikens, 2022; Mäkitie et al., 2016; Stürznickel et al., 2021). Another example is X-chromosomal *PLS3*, encoding for plastin 3. Mutations in this gene lead to complications raging from skeletal fragility in heterozygous females to severe childhood-onset osteoporosis in hemizygous males (Mäkitie et al., 2020).

However, the influence of genetic factors on BMD decreases with age. For instance, while genetic factors accounted for 58–88 % of BMD variation in pre-menopausal women, this proportion decreased to 37–54 % in post-menopausal women (Brown et al., 2005). This likely reflects a greater genetic impact on bone mass accrual rather than the rate of bone loss. Concurrently, controllable environmental factors gain more importance in predicting BMD with increasing age (Brown et al., 2005; Moayyeri et al., 2012). These include inadequate intake of essential nutrients, insufficient mechanical loading, smoking, substance use and use of oral contraceptives (Weaver et al., 2016b). Early management of these factors can substantially reduce OP risk later in life. In fact, it has been estimated that a 10 % increase in peak bone mass can reduce OP risk with 50 % in adults (Cummings et al., 1993), as well as delay OP onset with 13 years (Hernandez et al., 2003).

At a cellular level, aging in bone is characterized by a decrease in osteoblast number and activity, while osteoclasts become more active (Chung et al., 2014; Pietschmann et al., 2007). Moreover, age-related bone degradation is accelerated by accumulation of senescent bone cells (Kassem and Marie, 2011). Cellular senescence is a natural process in which cells lose their ability to divide and remain as viable but functionally altered cells, and is triggered by stressors including DNA damage, oncogene activation and inflammatory signals (Teissier et al., 2022b). Senescent cells in turn produce inflammatory markers to induce a cycle of bone remodeling, and can induce senescence in neighbouring cells. Accumulation of these cells can lead to bone-damaging chronic inflammation (Pignolo et al., 2021). Novel therapeutic agents aimed at clearing senescent bone cells are currently in development (Soto-Gamez and Demaria, 2017). Finally, osteocyte density and connectivity decline during aging (Qiu et al., 2002; Schurman et al., 2021), while the lacunae housing osteocytes diminish in number and undergo hypermineralization (Busse et al., 2010), together disrupting bone microstructural integrity and resulting in bone fragility.

Moreover, the skeletal system is not an isolated entity, but is interconnected with adjacent and remotely located tissues that communicate through the endocrine, nervous, immune, cardiovascular and muscular system (Gerosa and Lombardi, 2021; Imam et al., 2009; Karsenty and Olson, 2016; Lorenzo et al., 2008; Towler, 2008). These internal systems undergo changes with aging, thereby affecting bone remodeling dynamics. Finally, behavioural and environmental changes during aging require further adaptations of internal systems. In the following section, we delve into the primary risk factors for age-associated osteoporosis (AAOP) and explore how these can be targeted for prevention and treatment.

### 2.1 Mechanical loading

The human skeleton is built to bear the mechanical load that arises from daily physical activity. Static positions, i.e. sitting or standing, induce a baseline of mechanical loading that increases during dynamic movements, such as walking or lifting (Hart et al., 2017; Lanyon and Rubin, 1984). An individual's body weight and posture further determine the magnitude and direction of loading (Edmondston et al., 2011; Iwaniec and Turner, 2016). Notably, low BMI is an independent risk factor for OP due insufficient loading, aside from other factors as malnutrition and sarcopenia (Lee et al., 2020; Sieber, 2019; Tanaka et al., 2013).

Individuals tend to become less active as they age, with approximately one in four adults aged above 50 reported to have an inactive lifestyle in the United States (Watson et al., 2016). Common age-related physical changes, such as muscle weakness, joint stiffness and reduced balance make it more challenging to being active (Gomes et al., 2017). Furthermore, psychological factors as lack of motivation, social isolation and fear of falling can further discourage physical activity (Zhang et al., 2022). As a sedentary lifestyle does not provide enough loading to maintain bone and muscle mass, immobilization heightens susceptibility to fractures (LaMonte et al., 2019).

Osteocytes are central to the cellular response to mechanical loading by detecting bone deformation. In response, osteocytes modulate the release of various signaling molecules to stimulate bone remodeling, including nitric oxide, prostaglandin E2 and ATP (Bonewald, 2006; Han et al., 2004; Nicolella et al., 2006). Osteocytes promote bone formation in response to mechanical stress by suppressing sclerostin production, an inhibitor of the Wnt/β-catenin signaling in osteoblasts (Li et al., 2005; Robling et al., 2008), and by reducing the ratio of RANKL to RANK decoy receptor osteoprotegerin (OPG) production (Li et al., 2013; Zhang et al., 2015). The responsiveness of osteocytes to mechanical strain hinges on their interconnectivity. This is facilitated by an intricate web of dendritic extensions that foster intercellular communication (Klein-Nulend et al., 2012).

While regular exercise enhances peak bone mass during youth (Tveit et al., 2015), studies generally report only a maximum 1–3 % BMD increase in adults through regular exercise (Robling and Turner, 2009; Westcott, 2012; Zhao et al., 2015). Aside from age-related effects on osteoblast and osteoclasts, this may be explained by the age-related decline in osteocyte density and connectivity (Qiu et al., 2002; Schurman et al., 2021), which is further aggravated by long-term immobilization (Rolvien et al., 2020). Failing sclerostin inhibition has been proposed as an underlying molecular mechanism. In old mice, plasma sclerostin levels, and the expression of corresponding gene *Sost,* failed to decrease following exercise (Gardinier et al., 2018). Furthermore, and *Sost*-deficiency prevented mice from mechanical unloading-induced bone loss (Lin et al., 2009; Tatsumi et al., 2007). Clinical studies, however, report inconsistent results regarding sclerostin response to exercise in both young and old cohorts, thus requiring more data to clarify the role of sclerostin (Gombos et al., 2016; Janik et al., 2018).

Nonetheless, physical activity is widely recommended as a low-cost intervention to mitigate AAOP risk by, at least, preventing BMD loss. Given that an osteogenic response is generated by mechanical loading that exceeds the magnitude that is induced by normal daily activity, exercise should involve dynamic, novel and high magnitude loading. Accordingly, low-intensity exercises, such as walking, are shown not to improve BMD in older individuals (Ma et al., 2013; Nikander et al., 2010). Some, but not all, studies report that walking with added impact, such as brisk walking or hill walking, induce minor improvements in BMD (Ebrahim et al., 1997; Gába et al., 2016; Lan and Feng, 2022).

More favorable results are shown with resistance training, which is aimed at building muscular strength against external force. Kerr et al. (1996) showed a significant increase in BMD with resistance training focused on strength, but not with endurance-focused exercises. Peak load thus seems more important than the number of repetitions. Other studies favor higher load resistance training as well (Vincent and Braith, 2002; Zehnacker and Bemis-Dougherty, 2007). However, contradicting findings have also been published. A trial comparing low-load high-repetition to high-load low-repetition showed equal BMD improvements by both regimens. The authors noted that total load was similar in both groups, in contrast to earlier studies reporting a beneficial effect of high-loading (Bemben and Bemben, 2011). Another study reported that BMD loss could be mitigated with very high-repetition, low-intensity resistance training (Nicholson et al., 2015). Furthermore, two systematic reviews reported no BMD improvement, but only attenuation of BMD loss through high-intensity resistance training alone (Massini et al., 2022; Zhao et al., 2015). However, Zhao and colleagues did observe significant BMD improvements when resistance training was combined weight-bearing impact exercises, such as running and jumping (Zhao et al., 2015). Such high-intensity, multi-component programs are generally shown to improve BMD or at least mitigate BMD loss (Brooke-Wavell et al., 2022; Giangregorio et al., 2014; Kemmler et al., 2003; Martyn-St James and Carroll, 2010; von Stengel et al., 2011).

However, high-intensity exercise programs are limitedly recommended for osteoporotic individuals, due to concerns that these may exacerbate fracture risk (Giangregorio et al., 2014; Kohrt et al., 2004). The recent Lifting Intervention for Training Muscle and Osteoporosis Rehabilitation (LIFTMOR) trials addressed these concerns. They examined the efficacy and safety of high-intensity, progressive resistance and impact weight-bearing (HiRIT) in postmenopausal women and older men with low BMD (Harding et al., 2020; Watson et al., 2018). HiRIT involves large multi-joint compound movements such as squats and deadlifts, conducted in weight-bearing positions and recruiting extensive muscle groups. These exercises apply substantial loads at clinically relevant bone sites. In both trials, HiRIT significantly improved BMD at the lumbar spine and femoral neck, as well as bone geometry and functional performance, compared to the control group that did low-intensity exercise. Notably, both trials reported a high compliance rate and minimal adverse events.

Another consideration is the site-specificity of bone response to certain types of exercise. A Cochrane review concluded that high-intensity resistance, non-weight bearing exercise is most effective to improve femur neck BMD, while multi-component exercise programs are most effective at improving spine BMD (Howe et al., 2011). Aside from BMD, both low and high-impact training programs have been shown to improve balance and reduce fall risk (Sherrington et al., 2019).

Taken together, while no consensus is reached on the optimal type, duration and intensity of exercise for improving BMD in older adults, high-intensity resistance and multi-component programs targeted at relevant skeletal sites appear promising to improve BMD and fall risk in older and osteoporotic individuals.

### 2.2 Nutrition

Bone health relies on adequate supply of essential minerals, vitamins and protein. Mineralization (with calcium and phosphate) of newly formed -but still weak- bone, osteoid, is crucial to complete the process of bone formation. Aging is accompanied with alterations in nutrient absorption, utilization and metabolism. In addition, elderly tend to have diets that lack sufficient amounts of minerals and vitamins (Kelly et al., 2016). Resulting nutrient deficiencies have implications for skeletal integrity (Palacios, 2006).

Calcium is required for normal functioning of many biological processes including muscle contraction, nerve transmission and coagulation. Calcium levels are therefore strictly regulated, drawing upon the calcium reservoir in bone during low systemic calcium levels. Hypocalcaemia is a prevalent concern among elderly, particularly in hospital settings. A cross-sectional study including 400 elderly patients reported a 24 % prevalence, while a retrospective study found a 33 % prevalence among elderly hip fracture patients (Thapa and Rayamajhi, 2020; Wang et al., 2021). However, data on hypocalcaemia in non-institutionalized elderly is currently lacking.

The metabolism of calcium hinges on vitamin D levels. The main source of vitamin D3 is synthesis from 7-dehydrocholesterol through absorption of UV-B radiation in the skin (Lips, 2006). Additionally, vitamin D2 and D3 can be obtained through dietary sources and supplementation (Lamberg-Allardt, 2006). Vitamin D2 and D3 are metabolized by the liver into 25-hydroxyvitamin D, and further metabolized in the kidney into its active form 1,25-dihydroxyvitamin D by 1α hydroxylase. By acting on the vitamin D receptor (VDR), 1,25-dihydroxyvitamin D stimulates gastrointestinal absorption of calcium through induction of epithelial calcium channels and calcium and sodium-phosphate transporters (Moor and Bonny, 2016; Perez et al., 2008; Song et al., 2003). 1,25-dihydroxyvitamin may also directly act on bone cells, as osteoblast-specific VDR overexpression promotes bone formation and suppresses bone resorption (Eisman and Bouillon, 2014). The prevalence of vitamin D deficiency among non-institutionalized elderly reportedly ranges from 20 to 100 %, depending on geographical location (Holick et al., 2011). Deficiencies may result from diminished capacity to synthesize vitamin D3 from sunlight and declined ability to synthesize 1,25-dihydroxyvitamin D (Armbrecht et al., 1982; MacLaughlin and Holick, 1985; Tsai et al., 1984), as well as from decreased sunlight exposure. Furthermore, calcium absorption efficiency diminishes with increasing age (Avioli et al., 1965; Pattanaungkul et al., 2000), although underlying mechanisms require further elucidation (Fleet, 2022).

Low habitual dietary calcium intake as well as low serum 25-hydroxyvitamin D concentrations are associated with increased OP and fracture risk (Cauley et al., 2008; Warensjö et al., 2011). Given these consequences for bone health, adequate calcium and vitamin D intake are pivotal to mitigate AAOP risk. Studies traditionally report a reduced fracture risk though calcium and vitamin D supplementation (Chapuy et al., 1992; Jackson et al., 2006; Weaver et al., 2016a). Beneficial effects of vitamin D supplementation alone are controversial, with recent meta-analyses reporting conflicting results on the association between fracture risk and vitamin D supplementation (Kong et al., 2022; LeBoff et al., 2022).

Moreover, in a RCT among institutionalized older individuals with low 25-hydroxyvitamin D levels, increased dietary intake of high calcium and protein dairy foods was associated with a reduction of 33 % overall and 46 % hip fractures, and a decrease in fall risk of 11 % (Iuliano et al., 2021). Accordingly, adequate calcium and vitamin D dietary intake, as well as supplementation are part of the standard recommendations for aging adults (Nieves, 2003). Nonetheless, in the Netherlands, approximately 50 % of both men and women over the age of 50 do not meet the recommended intake of 1000 mg per day (van Rossum et al., 2020).

Finally, obesity is associated with lower circulating levels of 25-hydroxyvitamin D. This may be due to disposition sequestration of vitamin D in adipose tissue, thereby reducing its bioavailability (Ekwaru et al., 2014; Wortsman et al., 2000). Furthermore, vitamin D supplementation resulted in a significantly lower increase in 25-hydroxyvitamin D levels in overweight and obese individuals, as compared to participants with normal weight (Manson et al., 2019; Tobias et al., 2023). Obesity-related chronic low-grade inflammation and increased bone marrow adiposity may further harm bone health (Gkastaris et al., 2020). Obesity is a prevalent and growing concern in aging communities, with a prevalence of approximately 35 % in individuals above the age of 65 in the United States (Fakhouri et al., 2012), and a worldwide increase of 27.5 % of overweight individuals between 1980 and 2013 (Ng et al., 2014). While these data would advocate to adjust vitamin D supplementation according to body weight, obese individuals are also reported to have a lower threshold for parathyroid hormone (PTH) response to 25-hydroxyvitamin D levels. Although requiring more data, this suggests that lower 25-hydroxyvitamin D levels may have less implications for bone health as compared to non-obese individuals (Shapses et al., 2013).

Aside from calcium and vitamin D, dietary protein also supports bone health, with collagen serving as an essential component of bone and muscle tissue. Furthermore, protein intake is an important mediator of anabolic hormone insulin growth factor-I (IGF—I) release (Bonjour et al., 2001; Geusens and Boonen, 2002). In aged rats, protein diet diminishes IGF-I levels and bone formation rate (Bourrin et al., 2000). Similarly, a study in elderly patients showed a correlation between low protein diet and low circulating IGF-I levels, which could be mitigated by increased protein intake. Protein intake has also been shown to induce intestinal calcium uptake (Mangano et al., 2014), and urinary calcium excretion (Calvez et al., 2012). Some concerns have been raised that the calcium excreted in response to high dietary protein originates from bone. To clarify this, a clinical trial was conducted in which healthy adults received a low or high protein diet (Kerstetter et al., 2003). 80 % of increased urinary calcium originated from the extra absorbed calcium, implying that the remaining 20 % might originate from bone. Accordingly, some studies report increase in hip fracture under high protein diet (Frassetto et al., 2000), however others report an inverse trend (Wengreen et al., 2004). High protein intake also reduced the incidence of falls in an elderly cohort, likely due to increased bone and muscle strength (Zoltick et al., 2011). Nevertheless, due to the existing ambiguity in data, concrete dietary recommendations should be given with caution.

Furthermore, adequate and varied vegetable intake has been shown to be beneficial for bone health. In a cohort study among community driven elderly, high vegetable intake was associated with a reduced fall and fracture risk. Specifically, intake of cruciferous and allium varieties reduced fall risk (Sim et al., 2018). It is currently not well understood which nutrients explain the beneficial effects of vegetable intake on bone health. Webster et al. (2023) postulates a particular importance of nitrate and vitamin K1 due to their high concentration in cruciferous and allium vegetables and known beneficial effects on skeletal muscle. Dietary vitamin K intake indeed has been associated with reduced fracture risk in older hospitalized women (Sim et al., 2022), and postmenopausal women with osteoporosis (Huang et al., 2015), however effects on BMD are controversial (Vermeer, 2012). Furthermore, a recent meta-analysis reported no association between nitrate and fracture risks, and RCTs have reported conflicting effects of nitrate on BMD (Bolland et al., 2020; Jamal et al., 2013; Liu et al., 2022).

Finally, the balance between dietary fibre and fat content affects the uptake of nutrients. A diet high in fibre improves nutrient uptake and absorption by promoting a healthy gut microbiome and increasing gut motility (Adams et al., 2018). While dietary fat is required for absorption of the fat-soluble vitamin D, consuming high amounts of fat can slow down gut motility, impair nutrient absorption and promote inflammation (Youness et al., 2022).

Delivery of essential nutrients and oxygen to bone health relies on adequate blood perfusion, and cardiovascular function accordingly. Several clinical studies report an association between cardiovascular dysfunction and bone health. Yang and Huang (2023) reported a close relationship between cardiovascular disease (CVD) prevalence and low BMD in adults over 60 years of age, particularly within the femur. Furthermore, findings from a prospective cohort study indicated a higher CVD incidence in women with OP, and an association between OP and cardiovascular mortality in men (Rodríguez-Gómez et al., 2022). Dysfunctional vasculature is a common and growing concern in older adults, as it was estimated that cardiovascular disease will become the leading cause of death in individuals aged above 65 by 2030 (Heidenreich et al., 2011). Fortunately, several interventions aimed at improving bone health and cardiovascular health overlap, such as physical exercise and a well-balanced diet. Additionally, anti-resorptive drugs such as bisphosphonates have been suggested to cardiovascular complications, although robust RCT are warranted (Billington and Reid, 2020).

Altogether, maintaining a well-balanced diet, along with combined supplementation of vitamin D and calcium, have been shown to be effective in improving BMD score and reduce fracture risk within aging individuals (Boettger et al., 2018; Weaver et al., 2021; Weaver et al., 2016a). To improve adherence to lifestyle changes, nutritional education and intervention programs provide an effective measure. Several of these programs have been shown to increase vegetable, fruit and fibre intake (Neves et al., 2020).

### 2.3 Hormonal balance

Bone functions as an endocrine organ, supporting itself by producing e.g. bone-IGF, bone mineral protein (BMP), fibroblast growth factor 23 (FGF23), sclerostin and lipocalin. In addition, osteoblast-derived osteocalcin promotes insulin secretion and sensitivity. Several hormones, i.e. IGF and transforming growth factor-β (TGF-β), are stored within the bone matrix in their latent form. In bone regions undergoing resorption, these hormones are released and activated by osteoclasts to facilitate the remodeling process (Zhou et al., 2021).

#### 2.3.1 Glucocorticoids

Glucocorticoids (GCs) in physiological levels are essential for bone health especially during growth, primarily by stimulating osteogenesis from mesenchymal stromal cells at the expense of adipogenesis. In contrast, GCs in excess favor adipogenesis over osteogenesis, and induce a negative balance in bone remodeling. These imbalances lead to loss of bone mass and structure, and long-term synthetic GC use is associated with a high fracture risk of 30 % to 50 % (Angeli et al., 2006). GC excess induces an early, transient increase in bone resorption by extending osteoclast lifespan and suppressing osteoclast apoptosis, driven by an elevated RANKL/OPG ratio (Lee et al., 2021). With prolonged GC exposure, bone formation declines due to reduced osteoblast differentiation and proliferation, coupled with increased osteoblast apoptosis (Lee et al., 2021). This process is primarily mediated by stimulating inhibitors of the Wnt/β-catenin pathway (Frenkel et al., 2015). GCs are considered to mainly exert their effect by binding to the ubiquitously expressed glucocorticoid receptor (GR). GCs also signal through the high-affinity mineralocorticoid receptor (MR), which is expressed by osteoclasts, osteoblasts and osteocytes (Beavan et al., 2001). Although the role of the MR in bone remains to be elucidated, osteocyte-specific knockout of the MR attenuated loss of trabecular bone loss as induced by prednisolone (Fumoto et al., 2014).

The literature on age-related changes in cortisol levels, the endogenous GC variant in humans, is inconsistent, with studies reporting increases, decreases or no changes at all (Feldman et al., 2002; Rueggeberg et al., 2012; Seeman et al., 1997; Van Cauter et al., 1996). These discrepancies have been attributed to methodological limitations, and could also reflect a high interpersonal variability (Lupien et al., 1996). Notably, a recent longitudinal study among 1814 individuals aged 20 to 90 years old revealed a U-shaped pattern: cortisol levels decreased until the age of 20, stabilized, and then increased after 60 years old (Moffat et al., 2020). Nevertheless, more robust longitudinal data is needed to draw stronger conclusions. Furthermore, aging is suggested to be accompanied by blunted negative feedback response of GCs on adrenocorticotropic hormone (ACTH) release, and increased bone expression of 11β-hydroxysteroid dehydrogenase (11B-HSD) type 1, which converts inactive cortisone into the active hormone cortisol (Cooper et al., 2002). Aged mice showed increased adrenal production of corticosterone, the natural GC variant in rodents, and 11β-HSD type 1 expression in bone, with adverse effects on osteoblast and osteocyte apoptosis, bone formation rate and bone microarchitecture (Weinstein et al., 2010). In humans, serum cortisol concentration and bone loss rate are directly related in both sexes (Raff et al., 1999). Collectively, these studies suggest that maintaining adequate GC levels is crucial to mitigate AAOP risk. In addition to altered GC levels, aging is associated with a blunted amplitude in GC circadian rhythm (Bergendahl et al., 2000). Disrupted GC rhythm is detrimental to bone health as well, as explained in more detail in Section 2.7.

#### 2.3.2 Sex hormones

Sex hormones, estrogen and testosterone, are essential to maintain bone health in both men and women, with estrogen being most important (Falahati-Nini et al., 2000). Estrogen regulates turnover of trabecular bone in women, and cortical bone in both men and women (Bouillon et al., 2004; Khosla et al., 2012). According to a RCT among healthy elderly men, estrogen accounts for over 70 % of the effect of sex steroids in bone resorption (Falahati-Nini et al., 2000). Estrogen regulates both bone resorption and formation. It suppresses osteoclast differentiation and increases osteoclast apoptosis, predominantly through decreasing the RANKL/OPG ratio and blocking RANKL-signaling (Eghbali-Fatourechi et al., 2003; Hofbauer et al., 1999; Shevde et al., 2000). This is achieved by directly signaling through the estrogen receptor (ER) on osteoclasts, as osteoclast-specific ER deficiency is shown to reduce trabecular bone mass (Martin-Millan et al., 2010). Furthermore, estrogen represses osteoblast apoptosis and induces osteoblast differentiation. This is suggested to be achieved through increased BMP and Wnt/β-catenin signaling, and IGF-I production by osteoblasts (Almeida et al., 2007; Armstrong et al., 2007; Matsumoto et al., 2013). Notably, osteoblast linage-specific ER knockout models demonstrate loss of bone mass and poor response to mechanical loading in female mice (Määttä et al., 2013; Saxon et al., 2012). Finally, estrogen is required for osteocyte viability and mechanosensitivity (Emerton et al., 2010; Lee et al., 2003).

Testosterone is required for bone growth and maintenance in men, but also holds importance for women. Testosterone levels in women, produced by the adrenals, are positively associated with BMD, and low testosterone levels in women have been linked to OP (Zhang et al., 2022). Testosterone signals through the androgen receptor (AR), which is predominantly expressed on osteoblasts and osteocytes (Abu et al., 1997). It thereby stimulates bone formation by increasing osteoblast activity and lifespan, although prolonged exposure may inhibit osteoblast proliferation (Notelovitz, 2002). Furthermore, testosterone is metabolized into 17β-estradiol, the most potent form of estrogen. This is facilitated by aromatase, a specific component of cytochrome P450 that is expressed in osteoblasts and osteocytes (Gennari et al., 2004; Sasano et al., 1997). Variation in sex hormone levels is partly explained by environmental factors. In both men and women, higher BMI has shown an inverse relationship with both total testosterone levels and the testosterone/estradiol ratio (Meikle et al., 1989; Oztekin et al., 2020).

Sex hormone levels decline with age, with estrogen levels decreasing rapidly after menopause, and testosterone levels declining gradually over time (Horstman et al., 2012). This has consequences for bone, with estrogen deficiency being a pivotal cause of postmenopausal bone loss (Cheng et al., 2022). Given that men do not experience the same drastic decline in sex steroids as postmenopausal women do, AAOP risk is more affected by sex steroid deficiency in women than in men (Falahati-Nini et al., 2000). Aging is also accompanied by an increase in sex hormone-binding globulin concentration, which further reduces the amount of free testosterone and estrogen in the body (Aribas et al., 2021).

#### 2.3.3 Parathyroid hormone

In response to low circulating calcium and 1,25 hydroxyvitamin D levels, PTH is secreted from the parathyroid glands to increase bone resorption to release calcium that is stored within the bone matrix into the circulation turnover (Murthy and Duque, 2021). Specifically, PTH both stimulates RANKL and reduces OPG secretion, leading to increased bone resorption and loss of bone mass, mainly in the cortical area (Murthy and Duque, 2021). Continuously elevated PTH hormone levels have been directly associated with frailty (Murthy and Duque, 2021). Frailty represents a combination of factors that increase a person's vulnerability, of which OP is an important feature (Fried et al., 2001). Serum PTH levels increase with age in both men and women, however consequences for bone health differ (Murthy and Duque, 2021). For instance, elevated PTH levels with age have been reported to be correlated to increased bone resorption in elderly women but not men (Fatayerji and Eastell, 1999). This may be explained by the role of sex steroids in mitigating PTH effects on bone resorption (Falahati-Nini et al., 2000), and potentially by PTH receptor sensitivity differences. The age-related increase in PTH levels may reflect a physiological adaption to low circulating calcium and 1,25-dihydroxyvitamin D levels (Buchanan et al., 1988; Lips, 2001). Furthermore, glomerular filtration rate declines with age, and patients with declined renal function typically have elevated serum PTH levels, as discussed below (Noronha et al., 2022). However, the connection between renal function and age-related PTH elevation is not consistently reported. One study identified a decline in serum 25-hydroxyvitamin D levels as the best predictor of increased serum PTH levels, while they reported no relationship between renal function and PTH levels (Need et al., 2004). Another study similarly found no association with renal function, neither with serum 25-hydroxyvitamin D, ionized calcium, and phosphate levels (Carrivick et al., 2015). Variation in PTH levels is estimated to be 60 % determined by genetics (Hunter et al., 2001), and the responsiveness of various tissues to estrogen is also genetically controlled (Wall et al., 2014). Nonetheless, modifiable lifestyle factors as smoking, BMI, exercise, vitamin D, and calcium intake have been shown to influence PTH levels, and thus provide a potential target for intervention (Babic Leko et al., 2021).

#### 2.3.4 Growth hormone

Growth hormone (GH) stimulates the production of IGF—I. In addition to promoting bone growth during development, GH also helps maintain bone mass and structure in adulthood by stimulating osteoblast activity and inhibiting osteoclast activity (Olney, 2003). The secretion and response to GH and IGF-I decline with age, and this is suggested to contribute to age-related bone loss (Di Somma et al., 2011; Perrini et al., 2010).

#### 2.3.5 Dehydroepiandrosterone

Finally, aging is associated with reduced dehydroepiandrosterone (DHEA) levels, likely due to gradual shrinking of the zona reticularis in the adrenal gland (Yiallouris et al., 2019). DHEA has been shown to increase BMD, which may be ascribed to increased IGF-I expression and concurrent bone formation rate (Kirby et al., 2020). DHA supplementation could thus be postulated to improve bone health with aging.

#### 2.3.6 Chronic Kidney Disease

Furthermore, common metabolic diseases among older individuals, as Chronic Kidney Disease–Mineral and Bone Disorder (CKD-MBD), induce hormonal disbalances. Reduced renal function impairs the synthesis of 1,25 hydroxyvitamin D and disrupts phosphate metabolism, resulting in reduced intestinal calcium absorption and altered phosphate levels. This typically leads to elevated FGF23 levels and secondary hyperparathyroidism (Pazianas and Miller, 2021). Secondary hyperparathyroidism, in turn, leads to increased bone resorption, reduced BMD and increased OP risk (Bover et al., 2019; Hampson et al., 2021). However, the effects of impaired kidney function on bone health extend beyond hyperparathyroidism. For instance, CKD-related metabolic acidosis further exacerbates bone loss by stimulating bone resorption to buffer excess acid (Kim, 2021). As kidney function declines with age, CKD becomes increasingly prevalent, reportedly affecting 38 to 62 % in individuals aged above 70 years (Ebert et al., 2017).

#### 2.3.7 Mental stress

Hormonal balance can also be influenced by psychological factors such as mental stress. During prolonged stress, the hypothalamic-pituitary-adrenal (HPA) axis becomes deregulated, resulting in reduced GH levels and increased GC levels, consequently disrupting the balance in bone remodeling (Herman et al., 2016). Several studies in women report that higher perceived stress for a prolonged period, for example due to stressful work, is associated with a lower BMD and increased fracture risk (Kelly et al., 2019). This holds importance for older individuals, as aging is accompanied by biological and environmental changes that may pose stress on the individual. In particular, chronic conditions and pain induce mental stress in elderly (Vasunilashorn et al., 2015).

### 2.4 Metabolism

Metabolism comprises numerous biochemical and enzymatic reactions that are essential for homeostasis in organisms. The balance between anabolism and catabolism is constantly regulated and adapts to changes in energy expenditure (Moldakozhayev and Gladyshev, 2023). The cellular and systemic metabolic changes that occur during aging have consequences for bone health: metabolic disorders such as obesity and dyslipidaemia are associated with both aging and the development of various bone disorders (Asadipooya and Uy, 2019; Suzuki et al., 2020).

Lipid and bone metabolism are interconnected, as adipocytes and osteoblasts both derive from bone marrow stromal cells (BMSCs). The differentiation of BMSCs into either cell type depends on signaling cues and the microenvironment (Berendsen and Olsen, 2014). Aging is associated with an increase in bone marrow adiposity, resulting from a shift towards adipogenesis at the expense of osteogenesis (Ambrosi et al., 2017; Justesen et al., 2001). In addition, bone marrow adipocytes secrete bone-regulating factors including RANKL and OPG (Hu et al., 2021). Furthermore, adipocytes secrete factors that promote the conversion of osteoblasts towards an adipocyte-like cell. For instance, upregulated expression of adipocyte marker CD36 has been reported in osteoporotic women (Balla et al., 2008). Interestingly, another study found that expression of this marker was detected in osteoblasts of elderly subjects, but not in osteoblasts of younger subjects (Clabaut et al., 2021). The disruption of bone microenvironment by bone marrow adiposity is directly associated with fracture and OP risk (Veldhuis-Vlug and Rosen, 2018). In addition, several disorders that are associated with age, such as diabetes mellitus (DM) and obesity, both induce bone marrow adipogenesis and reduce bone formation (Ali et al., 2022).

Glucose metabolism is involved in bone health by maintaining an energy balance within bone cells. High glucose levels are linked to a decline in bone quality and a higher risk of fractures. Hyperglycemia negatively affects bone by reducing osteoblast metabolism and maturation (Eller-Vainicher et al., 2020). Furthermore, hyperglycemia promotes the formation of advanced glycation end products (AGEs), and AGE accumulation in bone has been shown to impair bone health by inducing osteoclastogenesis, reducing osteogenesis and impairing bone mineralization (Asadipooya and Uy, 2019). Furthermore, low levels of the anabolic hormone insulin, which are typical in uncontrolled DM type I, can reduce bone formation rate. In fact, mice with induced DM type I show a poor bone regeneration, which could be rescued by insulin supplementation (Cignachi et al., 2020). Since DM type I typically manifests during youth, individuals with the condition tend to have lower peak bone mass (Weber and Schwartz, 2016). This can accelerate the age at which the critical lower limit of bone mass is reached. Consequently, DM type I patients, who lack insulin, show increased hip fracture risk and decreased BMD. Paradoxically, individuals with DM type II, which mainly results from insulin insensitivity, also show elevated hip fracture risk, even though their BMD is significantly higher than non-diabetic individuals (Ma et al., 2012; Vestergaard, 2007). Thus, pathophysiological mechanisms for bone complications in DM type I and II differ. In a meta-analysis by Ma et al. (2012), young age, male gender, high BMI and poor glycemic control were all positively associated with high BMD in DM type II patients. Another meta-analysis by the same group revealed that poor glycemic control in diabetes type II patients was associated with an increased fracture risk, despite stronger bone geometry and increased BMD (Oei et al., 2013). Notably, while absolute BMD was increased in insulin-treated women with diabetes type II, this relationship was reversed when correcting for lean mass, suggesting a poor adaptation to mechanical loading (Garg et al., 2012).

In addition, individuals with DM may experience complications as nerve and muscle degeneration, and reduced vision due to retina degeneration. These complications may both directly affect bone health as well as increase the risk of falling (Gilbert and Pratley, 2015). To further complicate matters, antidiabetic drugs, such as thiazolidinedione, improve insulin sensitivity in patients, however also induce adiposity, bone loss and fracture risk (Lecka-Czernik, 2010; Yki-Jarvinen, 2004). Nevertheless, DM is reportedly more strongly related to OP risk in a younger population, presumably as other OP risk factors are less prevalent at a younger age (Lin et al., 2021).

### 2.5 Brain and nervous system

Cognitive decline is directly associated with an increased fall risk, and with falling-induced fracture risk (Tsutsumimoto et al., 2018). Aside from this evident association between bone and cognitive health, both are involved in similar hormonal, immune and molecular pathways, and share common risk factors for disease (Kelly et al., 2020). The brain regulates bone metabolism by sensory innervation and endocrine cross-talk between brain and bone (Masi, 2012). In this regard, neurological disorders may mediate secondary effects in bone, while bone disturbances have also been reported to precede cognitive decline (Xiao et al., 2023). For instance, expression of several Alzheimer's disease-related genes was associated with elevated *RANKL* and tartrate-resistant acid phosphatase (*TRAP*) gene expression, as well as reduced femoral cortical thickness (Stapledon et al., 2021). Another example of brain-bone crosstalk is neuropeptide Y (NPY), which is expressed by central and peripheral nervous systems to regulate bone formation in response to obesity and fasting (Botelho and Cavadas, 2015). NPY expression is increased during “starving” conditions to conserve energy, however also reduces bone formation. During adequate nutrient intake, NPY levels are low and thereby allow normal bone remodeling. Notably, NPY levels increase with aging and has been linked to several aging hallmarks including cellular senescence (Botelho and Cavadas, 2015). The connection between cognitive conditions and OP is still an active area of investigation and not widely recognized within clinical practice. This is reflected by the fact that less than 5 % of hip fracture patients with dementia are referred for treatment of OP, compared to around 30 % of hip fracture patients within the overall population (Bliuc et al., 2021), although this may also be explained by a low life expectancy.

### 2.6 Inflammation

Pro-inflammatory cytokines significantly contribute to bone loss while simultaneously increasing susceptibility to osteoporotic fractures (Amarasekara et al., 2015). During aging, the immune system experiences changes that are similar as seen during chronic stress. Aging induces immunosenescence, with elevated serum levels of acute phase proteins and pro-inflammatory cytokines (Teissier et al., 2022a). Furthermore, periodontitis has been shown to contribute to chronic low-grade inflammation and is highly prevalent in older adults, affecting up to 44 % of individuals aged above 65 (Nazir, 2017). Studies have demonstrated that pro-inflammatory cytokines, such as tumor necrosis factor alpha (TNF-alpha), interleukin 6 (IL-6) and IL-12, amplify bone loss (Kany et al., 2019). Notably, higher baseline inflammatory marker levels were associated with higher fracture risk in a large population study (Cauley, et al., 2007). Furthermore, wear particles from prosthetic devices, such as knee and hip replacements, can induce local inflammation and subsequent bone degeneration (Ingham and Fisher, 2005). The process of bone resorption also triggers release of inflammatory cytokines thereby establishing a vicious cycle of inflammation and bone loss (Amarasekara et al., 2015). The relationship between inflammation and OP is well exemplified by rheumatoid arthritis (RA). In RA, both joints and bone deteriorate due to the release of metalloproteinases and proinflammatory cytokines (IL-1, TNF-α) that lead to cartilage and bone damage (Zwerina et al., 2007). Consequently, the severity of the disease acts as distinct risk factor for OP in individuals diagnosed with RA.

### 2.7 Circadian rhythm

An often unrecognized contributing factor to AAOP is the disruption of circadian rhythm. Circadian rhythm plays a significant role in bone health, and disruptions to this rhythm, such as those caused by shift work, elevate the risk of OP (Schilperoort et al., 2020; Smit et al., 2022; Winter et al., 2021). At the core of internal circadian regulation is the suprachiasmatic nucleus (SCN), whose output is synchronized to internal and external stimuli such as light/dark rhythm.

As individuals age, amplitude and phase of rhythmic SCN output becomes disrupted (Nakamura et al., 2016). These disturbances are likely due to age-related changes within the SCN (Biello, 2009; Nakamura et al., 2011). Behavioural rhythms, such as sleep/wake cycles, also become disrupted with increasing age (Duffy et al., 1998; Mattis and Sehgal, 2016). Notably, implantation of foetal SCN tissue in aged rats could restore some rhythmic behaviours, implying a causal link between SCN aging and behavioural arrhythmia (Cai et al., 1997; Li and Satinoff, 1998). Consequently, when behavioural rhythms decline, the SCN no longer receives sufficient rhythmic input. This may be compounded by a compromised sensitivity and exposure to light (Sletten et al., 2009).

Several hormones that regulate bone remodeling exhibit a diurnal rhythm, including PTH, estrogen, testosterone and GCs, and disturbing rhythmic signaling of these factors in bone has negative consequences for bone health (Bergendahl et al., 2000; Sletten et al., 2009; Smit et al., 2022). For instance, flattening of the diurnal amplitude in GC levels, without increasing overall levels, was shown to induce an osteoporotic phenotype in female mice (Schilperoort et al., 2021; Winter et al., 2021). Notably, circadian rhythm also influences the adaptation of bone to mechanical loading in animal models, emphasizing the intricate interplay between circadian rhythm and bone health (Bouchard et al., 2022).

## 3 AAOP prevention and treatment strategies

Interventions for AAOP can be used with the aim of prevention, disease management and symptom relief. Primarily, adopting a healthy lifestyle throughout life enables achieving an optimal peak bone mass and preservation of bone mass and structure during aging. These behaviours include regular physical activity, a balanced diet, moderation of alcohol consumption and smoking cessation, as discussed in previous sections. Specifically in regards to offset age-related changes in nutrient uptake and metabolism, it is advisable to enhance calcium and vitamin D intake, either through dietary sources or supplements. Standard guidelines suggest the initiation of vitamin D supplementation after menopause, or at the age of 70 for men (Voulgaridou et al., 2023).

Aside from preserving bone quality, fracture risk in elderly can be effectively mitigated through fall prevention strategies. These include creating a safe living environment with limited tripping hazards and assistive devises such as a stairlift (Campani et al., 2021). Furthermore, physical exercise has been shown to effectively improve balance and reduce fall rates in elderly, as discussed in Section 2.1 (Papalia et al., 2020).

To the extension of lifestyle modifications, a range of anti-osteoporotic drugs are currently utilized, comprising both antiresorptive and bone-anabolic medications. Bisphosphonates are typically the first drug of choice. These antiresorptive bisphosphonates adsorb to hydroxyapatite crystals, especially in areas of high bone remodeling. Upon bone resorption, osteoclasts internalize the bisphosphonate molecules, leading to reduced osteoclast function or apoptosis, depending on the subtype (Jobke et al., 2014; Rodan and Fleisch, 1996). Another antiresorptive agent also particularly recommended for postmenopausal osteoporosis is denosumab. Similar to OPG, denosumab acts as a human monoclonal antibody against RANKL, thereby acting on all phases of osteoclasts (Kostenuik et al., 2009). While improving BMD during treatment, discontinuation leads to increased bone resorption and BMD loss. The mechanisms underlying this rebound effect are an active area of research. McDonald et al. (2021) suggests that denosumab induces the fission of osteoclasts, and these accumulated ‘osteomorphs’ collectively fuse into active osteoclasts after discontinuation. Fu et al. (2023) proposes an alternative hypothesis, asserting that denosumab treatment disrupts the OPG/RANKL balance due to reduced osteoblast and osteocyte formation. The latter hypothesis is supported by recent clinical trials in which BMD gain with denosumab could be maintained by concurrent treatment with bone-anabolic romosozumab (Ebina et al., 2020; Kendler et al., 2019).

Medications that stimulate bone formation include teriparatide and romosozumab. Teriparatide is a recombinant form of human PTH, that stimulates new bone formation when administered in an intermittent regime (Yamamoto et al., 2016). While chronically elevated PTH levels stimulate bone resorption and thereby result in bone loss, pulsatile PTH peaks can induce a rapid increase in bone formation by inhibiting osteoblast apoptosis and promoting osteoblast formation (Silva et al., 2011). This is explained by the difference in lifespan of osteoclasts and osteoblasts; a few days versus three months respectively (Dobnig et al., 2005). Romosozumab is a monoclonal antibody targeting sclerostin (McClung et al., 2014). Romosozumab has been shown to induce bone formation and reduce bone resorption, and is currently prescribed to post-menopausal women with a low BMD score and vertebral fractures (Kompas; McClung et al., 2014). Given that mechanical loading suppresses sclerostin secretion, romosozumab has been proposed as a therapeutical option for older individuals that are immobilized for a prolonged period (Rolvien et al., 2020). However, caution is needed due to potential cardiovascular adverse events (Saag et al., 2017). Importantly, pharmacological treatments have also been shown to be safe and effective in the ‘oldest old’, i.e. individuals aged above 80 (Vandenbroucke et al., 2017).

Given the diurnal patterns in both bone turnover and its regulators, timing the administration of therapeutic agents, referred to as chronotherapy, can be effective in enhancing treatment outcomes and mitigating side effects (Winter et al., 2021). For example, teriparatide demonstrates heightened effectiveness when administered in the morning, as evidenced by lower bone resorption marker CTx values and a more substantial increase in lumbar spine BMD, as compared to evening administration (Luchavova et al., 2011; Michalska et al., 2012). Moreover, administering vitamin D3 at the onset of the dark phase in aged rats, as opposed to within the light phase, resulted in a greater BMD increase and reduced the severity of side effects, including hypercalcemia (Tsuruoka et al., 2001).

While medical treatments and facilitated prevention programs can promptly improve bone health and reduce fracture risk, continuous management of AAOP risk factors is necessary to maintain bone health throughout all of adulthood. Therefore, greatest gains can be achieved through adequate self-management, which requires sufficient health literacy. This can improve the low adherence to AAOP intervention programs and medication, as individuals need to comprehend the reasons and methods for adhering to their therapies. In addition to proper patient education, treatment options with minimal adverse effects and lower frequent dosing have been reported to enhance therapy adherence (Hiligsmann et al., 2019).

Scenario 2 **Mechanical loading after menopause**

Mrs. Lee is a postmenopausal 60-year-old woman, diagnosed with vitamin D deficiency. Mrs. Lee also has a family history of OP. Her genetic predisposition means that she is at a higher risk of developing osteoporotic fractures, even with adequate hormone levels and exercise. Additionally, her previous knee injury makes it difficult for her to engage in, which is considered to be most effective type of physical exercise to stimulate bone formation. Mrs. Lee is concerned about her bone health and wants to know what she can do to prevent OP fractures.

To address these challenges, Mrs. Lee's healthcare primarily recommends to increase her vitamin D and calcium intake through a balanced diet and supplementation. Furthermore, she is advised to engage in low-impact weight-bearing exercises, such as brisk walking, and resistance training with weights or resistance bands. Initially of moderate impact and building towards higher intensity according to physical capabilities. This tailored exercise program may preserve bone mass without placing excessive strain on her joints (Nikander et al., 2010). The combination of mechanical and nutritional intervention allows Mrs. Lee to proactively reduce her osteoporotic fracture risk. In addition, given her postmenopausal status and family history of OP, Mrs. Lee is referred for a DXA scan to determine her BMD, together with a vertebral fracture assessment (VFA) to detect spinal fractures.

Alt-text: Scenario 2

## 4 Integrating risk factors to personalize AAOP treatment

Physical inactivity, malnutrition, hormonal and metabolic disbalance, cognitive decline, (chronic) inflammation and blunted circadian rhythm are all well-recognized as individual contributing factors to AAOP. However, AAOP is not a mere sum of individual factors, but a complex integration of these factors on biological, systemic and behavioural level, as exemplified in patient scenario 2.

As a means to integrate individual risk factors to accurately predict AAOP risk, several assessment tools have been developed. These tools are aimed at predicting increased fracture risk and typically integrate factors as BMD, age, body weight, history of fractures and use of medication that list OP as adverse effect (Rubin et al., 2013). As the effect of BMD on fracture risk in itself is affected by the presence of other risk factors, fracture risk assessment tools can also assist in deciding which patients should be referred for BMD measurement (Compston et al., 2009). The Fracture Risk Assessment Tool (FRAX) is frequently used, which includes 10 clinical risk factors with or without BMD to predict the 10-year probability of osteoporotic fractures (Kanis et al., 2007). Fracture risk can also be determined by an online tool developed by Garvan institute, which performs similar to FRAX but also includes fall risk which is of particular importance for the elderly population, and is freely accessible (Agarwal et al., 2022). Interestingly, a review on AAOP risk tools concluded that the more complex FRAX performed similarly to simpler screening tools, or even age alone (Rubin et al., 2013). Other studies also report that increasing the complexity of tools does not improve prediction accuracy (Leslie et al., 2016). One may conclude that taking account of all factors that contribute to AAOP is not necessary to accurately identify individuals at risk, and even a routine screening at a certain age may be most effective. However, there is a lack of high-quality studies that have evaluated their effectiveness in selecting patients for therapy and improving fracture outcomes (Rubin et al., 2013).

Identifying individuals at risk is only the starting point for the management of AAOP. In fact, the effectivity and durability of interventions highly depends on the precise combination of risk factors, which can vary for each individual. Therefore, a personalized approach that takes into account the unique circumstances of the individual may result in the most effective care for AAOP. This is in line with the growing support in literature for personalized medicine in OP care.

## 5 Conclusion

In conclusion, AAOP presents a global health challenge that becomes increasingly relevant in our aging society. This review has highlighted the multifaceted nature of AAOP, discussing the numerous biological and behavioural changes that contribute to its development and guide treatment decisions. As every patient presents a unique disease etiology, the management of AAOP can benefit from holistic and patient-centred approach. This entails integration of lifestyle changes, fall prevention strategies, and medical therapy. Tailoring interventions to the specific circumstances and needs of individual patients will maximize outcomes and improve overall disease management. Moving forward, the development of an integrated disease model for AAOP that encompasses all significant interactions between various factors would be instrumental in designing comprehensive and personalized treatment strategies. Nevertheless, increased awareness of the diverse factors influencing disease progression can already guide healthcare professionals in earlier detection and improved management of AAOP.

## CRediT authorship contribution statement

**A.E. Smit:** Writing – review & editing, Writing – original draft, Visualization, Validation, Resources, Project administration, Methodology, Formal analysis, Conceptualization. **O.C. Meijer:** Writing – review & editing, Writing – original draft, Validation, Supervision, Resources, Project administration, Methodology, Formal analysis, Conceptualization. **E.M. Winter:** Writing – review & editing, Writing – original draft, Validation, Supervision, Resources, Project administration, Methodology, Funding acquisition, Formal analysis, Conceptualization.

## Declaration of competing interest

The authors declare that they have no known competing financial interests or personal relationships that could have appeared to influence the work reported in this paper.

## Data Availability

No data was used for the research described in the article.
